# Aspalathin Reverts Doxorubicin-Induced Cardiotoxicity through Increased Autophagy and Decreased Expression of p53/mTOR/p62 Signaling

**DOI:** 10.3390/molecules22101589

**Published:** 2017-09-22

**Authors:** Rabia Johnson, Samukelisiwe Shabalala, Johan Louw, Abidemi Paul Kappo, Christo John Frederick Muller

**Affiliations:** 1 Biomedical Research and Innovation Platform (BRIP), South African Medical Research Council (MRC), Tygerberg 7505, South Africa; samukelisiwe.shabalala@mrc.ac.za (S.S.); johan.louw@mrc.ac.za (J.L.); christo.muller@mrc.ac.za (C.J.F.M.); 2 Division of Medical Physiology, Department of Biomedical Sciences, Faculty of Medicine and Health Sciences, Stellenbosch University, Tygerberg 7505, South Africa; 3 Department of Biochemistry and Microbiology, University of Zululand, Kwadlangezwa 3886, South Africa; kappoA@unizulu.ac.za

**Keywords:** autophagy, cardiotoxicity, doxorubicin, aspalathin, oxidative stress, apoptosis, cardiomyopathy

## Abstract

Doxorubicin (Dox) is an effective chemotherapeutic agent used in the treatment of various cancers. Its clinical use is often limited due to its potentially fatal cardiotoxic side effect. Increasing evidence indicates that tumour protein p53 (p53), adenosine monophosphate-activated protein kinase (AMPK), nucleoporin p62 (p62), and the mammalian target of rapamycin (mTOR) are critical mediators of Dox-induced apoptosis, and subsequent dysregulation of autophagy. Aspalathin, a polyphenolic dihydrochalcone *C*-glucoside has been shown to activate AMPK while decreasing the expression of p53. However, the role that aspalathin could play in the inhibition of Dox-induced cardiotoxicity through increased autophagy flux remained unexplored. H9c2 cardiomyocytes and Caov-3 ovarian cancer cells were cultured in Dulbecco’s Modified Eagle’s medium and treated with or without Dox for five days. Thereafter, cells exposed to 0.2 µM Dox were co-treated with either 20 µM Dexrazozane (Dexra) or 0.2 µM aspalathin (ASP) daily for 5 days. Results obtained showed that ASP mediates its cytoprotective effect in a p53-dependent manner, by increasing the Bcl-2/Bax ratio and decreasing apoptosis. The latter effect was diminished through ASP-induced activation of autophagy-related genes (Atgs) with an associated decrease in p62 through induction of AMPK and Fox01. Furthermore, we showed that ASP was able to potentiate this effect without decreasing the anti-cancer efficacy of Dox, as could be observed in Caov-3 ovarian cancer cells. Taken together, the data presented in this study provides a credible mechanism by which ASP co-treatment could protect the myocardium from Dox-induced cardiotoxicity.

## 1. Introduction

Tumour suppressor protein (p53), also known as the cellular gatekeeper, is a phospho protein that is activated in response to various stresses including DNA damage, oxidative stress, chronic starvation (nutrient deprivation), endoplasmic reticulum stress and hypoxia [1]. Once activated, p53 orchestrates a signaling cascade that induces the initiation of apoptosis and autophagy.

Apoptosis and autophagy are important cellular processes known to play a central role in the maintenance and homeostasis of various biological signals. While apoptosis accomplishes its role through programmed cell death, autophagy maintains cellular homeostasis through the segregation and delivery of damaged intracellular organelles and molecules to the lysosome machinery for proteolytic degradation [2,3,4]. The machinery includes activation of various autophagy-related genes (Atgs). Once activated, Atgs complexes with the non-soluble microtubule-associated protein 1 light chain 3 (LC3) to increase autophagic flux through decreased expression of sequestosome 1 (p62/SQSTM1) [5].

Autophagy can block induction of apoptosis, as an effective link exists between apoptosis and autophagy. Under certain conditions, autophagy constitutes an adaptive response in order to avoid cell death and through this subdues apoptosis [4,6]. This, process is triggered by common upstream molecular switches such as the p53/adenosine monophosphate—activated protein kinase/mammalian target of rapamycin (p53/AMPK/mTOR) pathway, a regulatory network within which a complex interplay exists between autophagy and apoptosis. However, the intricate interplay and activation of either a cell death or cytoprotective response depends on the environmental stress at hand.

During increased oxidative stress or nutrient starvation, autophagy is rapidly upregulated to restore metabolic homeostasis while inhibiting apoptosis, thus facilitating survival under stressful conditions. One such stress is Doxorubicin-induced cardiotoxicity. Doxorubicin (Dox) is a first line anti-cancer chemotherapeutic drug used in the treatment of various adult and pediatric cancers [7]. Since its introduction in the early 1970s, cancer survival rates have increased [8]. However, this increase in cancer survival was concomitant to an increase in cardiac dysfunction, as the efficacy of Dox has been hindered by its acute and chronic dose-dependent cardiotoxicity side effect [9,10].

Doxorubicin is known to induce cardiotoxicity via a p53-dependant increase in apoptosis and decreased mTOR-dependent regulation of autophagy [11]. The latter cellular processes are regulated by a central checkpoint, AMPK [12]. A study done by Wang and colleagues (2012) showed that AMPK inhibition by Dox resulted in an increased accumulation of p53 expression and subsequent cell death, while pharmacological activation of AMPK alleviated this effect [12]. Similarly, Lui (2015) showed t Dox-induced apoptosis in an AMPK/p53 dependant manner. Treatment with resveratrol was able to ablate this effect through increased phosphorylation of AMPK [13]. Thus, a complex interplay exist between p53, AMPK, and mTOR, and activation of these processes can either drive or inhibit autophagy and apoptosis.

Of note, Dexrazoxane (Dexra) an established cardio protectant is the only Food and Drug Administration (FDA) approved drug used in combination with Dox. Furthermore, one of the mechanisms by which Dexra protects the heart from Dox is by chelating free iron, thereby reducing reactive oxygen species (ROS) production. However, in a study performed by Dickey and colleagues, the authors showed that apart from reducing ROS, Dexra was able to increase the pro-survival autophagy marker, LC3 II, and decrease caspase 3 activity, when compared to Dox treatment, in the hearts of spontaneous hypertensive rats [14]. The observed effect was independent of Dox treatment, making Dexra an ideal cardioprotective agent to alleviate Dox cardiotoxicity in cancer patients. However, Dexra has been shown to induce secondary malignancies [15], and thus alternative treatment strategies are currently being investigated with lower or no toxic side effects.

Of note, Aspalathin (ASP), a dihydrochalcone *C*-glucoside, is a polyphenolic compound unique to *Aspalathus linearis* and has been extensively investigated for its antioxidant, anti-inflammatory, and anti-apoptotic properties [16,17,18]. Aspalathin is a known activator of p53, AMPK, and mTOR. Previous studies from our group confirmed that ASP was able to protect the myocardium from high glucose-induced myocardial apoptosis by increasing the Bcl-2/Bax ratio, subsequently hindering the release of cytochrome C from mitochondria and inhibiting caspase 3 activation [16]. In addition, we propose that aspalathin is able to activate autophagy through increased expression of the master metabolic regulator and energy sensor, AMPK. This study set out to investigate if ASP can inhibit Dox-induced cardiotoxicity through increased AMPK and ATG expression while decreasing the expression of p53 signaling and thus prevent Dox-induced cardiomyocyte apoptosis of H9c2 cells.

## 2. Results

### 2.1. The Effect of ASP Treatment on ATP Activity as a Measurement of Metabolic Activity in H9c2 Cells

**Figure 1:**
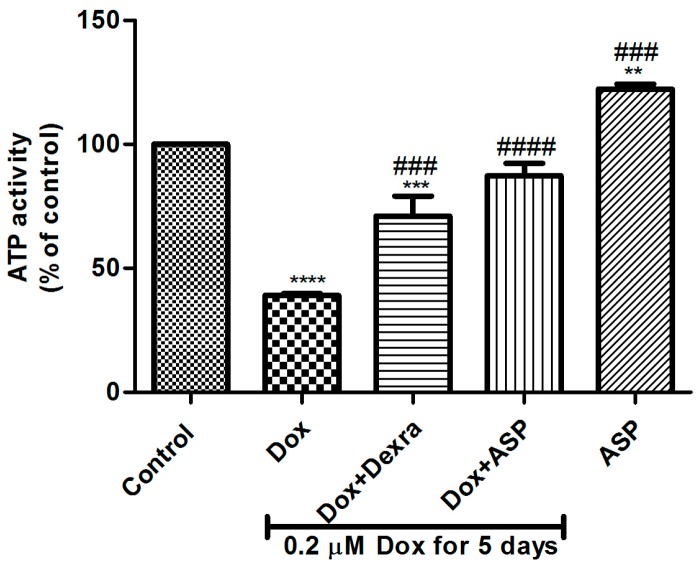
The effect of aspalathin (ASP) co-treatment on metabolic activity of H9c2 cells exposed to Doxorubicin. H9c2 cells were treated with 0.2 µM doxorubicin (Dox) alone (positive control) or co-treated in combination with either 20 µM dexrazoxane (Dexra) or 0.2 µM ASP daily for 5 days. Control samples were either exposed to media only or 0.2 µM ASP for 5 days. Results are expressed as the mean ± SEM of three independent biological experiments, each with three technical replicates (*n* = 9). *** *p* ≤ 0.001, **** *p* ≤ 0.0001 versus control and ^###^ *p* ≤ 0.001, ^####^ *p* ≤ 0.0001 versus Dox.

To examine the effect of Dox on cytotoxicity, H9c2 cells were co-treated with either 0.2 µM ASP or 20 µM Dexra daily for 5 days. Analysis of results showed that Dox treatment at a dose of 0.2 µM, daily for 5 days, significantly decreased ATP activity (39 ± 0.7%, *p* ≤ 0.0001) when compared to the normal control (100%) (Figure 1). Dox co-treatment with either Dexra (71 ± 8.021%, *p* ≤ 0.001) or ASP (87.33 ± 5.01%, *p* ≤ 0.0001), significantly ablated this effect when compared to the Dox control group. Interestingly, treatment of H9c2 cells with 0.2 µM ASP for 5 days resulted in an increased metabolic activity when compared to the normal control (122 ± 1.9%, *p* ≤ 0.01 compared to 100%) and Dox treatment control (122 ± 1.9%, *p* ≤ 0.001 compared to 39%).

### 2.2. ASP Decrease Caspase 3/7 Activity and Reverts Dox-Induced Inhibition of Autophagy in H9c2 Cells

#### 2.2.1. The Effect of ASP on p-AMPK Expression

**Figure 2:**
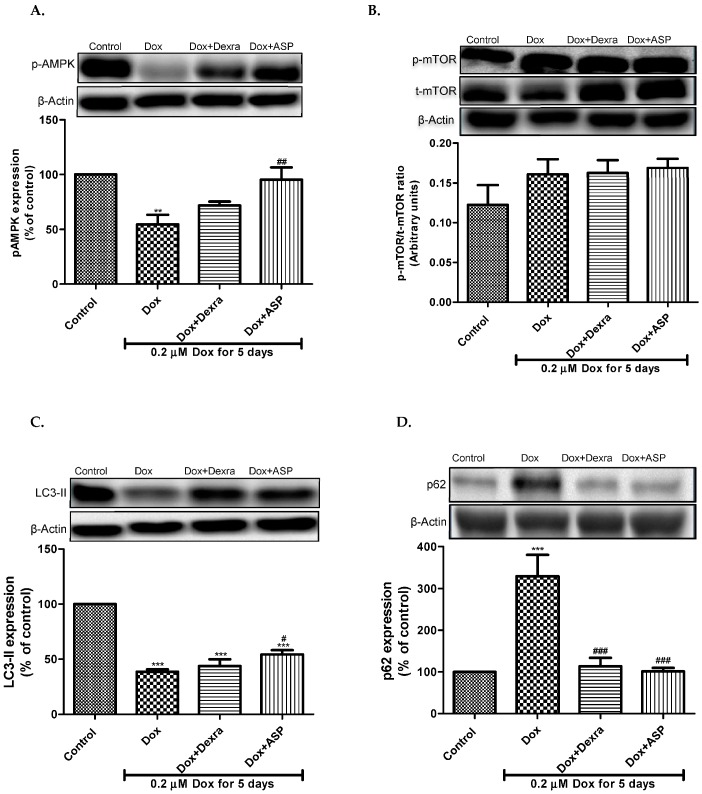
The effect of aspalathin on autophagy proteins. (**A**) Represents the expression of phosphor (p)adenosine monophosphate-activated protein kinase (AMPK); (**B**) the expression of mammalian target of rapamycin (mTOR); (**C**) the expression of LC3-II; and (**D**) the expression of p62 in doxorubicin (Dox)-induced cardiotoxicity. H9c2 cells were treated with 0.2 µM Dox alone or in combination with either 20 µM dexrazoxane (Dexra) or 0.2 µM ASP daily for 5 days. Control samples were exposed to media only. All blots were normalized to beta actin. Results are expressed as the mean ± SEM of three independent biological experiments, each with three technical replicates (*n* = 9). ** *p* *** *p* ≤ 0.01 versus control and ^#^ *p* ≤ 0.05, ^##^ *p* ≤ 0.01 and ^###^ *p* ≤ 0.01 versus Dox.

Dox-induced AMPK suppression was known to result in decreased autophagy. In this study, the effect of ASP on phosphor(p)-AMPK activation was investigated. A decrease in p-AMPK expression was observed after H9c2 cardiomyoblasts were treated with 0.2 µM Dox for 5 days (from 100% to 54.56 *±* 8.8%, *p* ≤ 0.01), indicating that Dox treatment inhibited p-AMPK expression. However, co-treatment with Dox + ASP was able to significantly increase p-AMPK expression by approximately 41% (95.41 *±* 11.2%, *p* ≤ 0.01) when compared to the Dox treated control. Similarly, an increased p-AMPK activity was observed when H9c2 cells were co-treated with Dox + Dexra (71.82 *±* 3.5%), though not significant (Figure 2A).

#### 2.2.2. The Effect of ASP on the Expression of mTOR

mTOR is a protein kinase and key inhibitor of autophagy, a process that contributes to cell survival. To establish the role of mTOR signaling in Dox-induced cardiotoxicity, the ratio of p-mTOR/t-mTOR was determined. A slight increase in the p-mTOR/t-mTOR ratio (0.161 ± 0.02) was observed upon Dox treatment when compared to the normal control group (0.12 *±* 0.03)*.* Co-treatment of Dox with either Dexra or ASP were unable to ablate this response (0.16 ± 0.02 or 0.16 ± 0.01, respectively) (Figure 2B).

#### 2.2.3. The Effect of ASP on the Expression of LC3-II

In addition, LC3-II is a known biomarker for autophagy as it initiates the formation of the autophagosome. In this study, the expression of LC3-II was evaluated using Western blot analysis. Results obtained showed that Dox treatment decreased expression of LC3-II (38.71 ± 2.1%, *p* ≤ 0.001), suggesting the inhibition of autophagy upon Dox treatment when compared to the untreated control group (100%) (Figure 2C). Interestingly, Dox + ASP moderately increased the expression of LC3-II (54.33 ± 3.9%, *p* ≤ 0.05) when compared to Dox control group while co-treatment with Dox + Dexra did not have an effect on LC3-II expression (43.86 ± 6.07%).

#### 2.2.4. The Effect of ASP on the Expression of p62

p62 is a well-known marker for autophagy and its expression levels are known to be inversely proportional to the degree of autophagy induction. In the present study, our results showed that Dox treatment resulted in an increased expression of p62 (329.6 ± 50.99, *p* ≤ 0.001) when compared to the normal control group (100%). Co-treatment of Dox with either ASP or Dexra was able to decrease this response significantly (113 ± 20.4, *p* ≤ 0.001 or 101.4 ± 8.2, *p* ≤ 0.001, respectively) (Figure 2D).

#### 2.2.5. The Effect of ASP on p53 Expression

**Figure 3:**
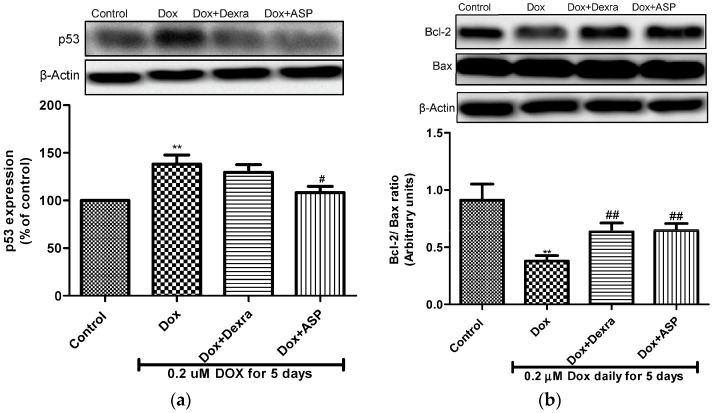
(**a**) The effect of aspalathin (ASP) on apoptosis proteins. (**a**) Represents the expression of p53 and (**b**) the expression of the Bcl-2/Bax ratio. H9c2 cells were treated with 0.2 µM Dox alone or in combination with either 20 µM dexrazoxane (Dexra) or 0.2 µM ASP daily for 5 days. Control samples were exposed to media only. Results are expressed as the mean ± SEM of three independent biological experiments, each with three technical replicates (*n* = 9). ** *p* ≤ 0.01 versus control and ^#^ *p* ≤ 0.05, ^##^ *p* ≤ 0.01 versus Dox.

Alterations in p53 protein expression induced by Dox treatment were analysed using Western blot analysis. The results obtained demonstrated that Dox treatment alone significantly increased the expression of p53 (138.1 ± 9.7%, *p* ≤ 0.01), when compared to untreated cells. Co-treatment with ASP resulted in a significant decrease in the expression of p53 (108.2 ± 6.5%, *p* ≤ 0.05) when compared to Dox-treated cells. A combination of Dox and Dexra decreases p53 expression, but not significantly (129.6 ± 7.9%) (Figure 3A).

#### 2.2.6. The Effect of ASP on the Bcl-2/Bax Ratio

Suppression of the Bcl-2/Bax ratio has been associated with increased caspase-3 activity and increases apoptosis. In this study, a significant decrease in the Bcl-2/Bax ratio was observed when cells were exposed to Dox when compared to the normal control (0.38 ± 0.05, *p* ≤ 0.01 compared to 0.9 ± 0.14). Interestingly, co-treatment with either ASP or Dexra significantly increased the Bcl-2/Bax ratio (0.65 ± 0.06, *p* ≤ 0.01 or 0.61 ± 0.08, *p* ≤ 0.01) when compared to Dox treatment alone (Figure 3B).

#### 2.2.7. The Effect of ASP on Capase-3/7 Activity

**Figure 4:**
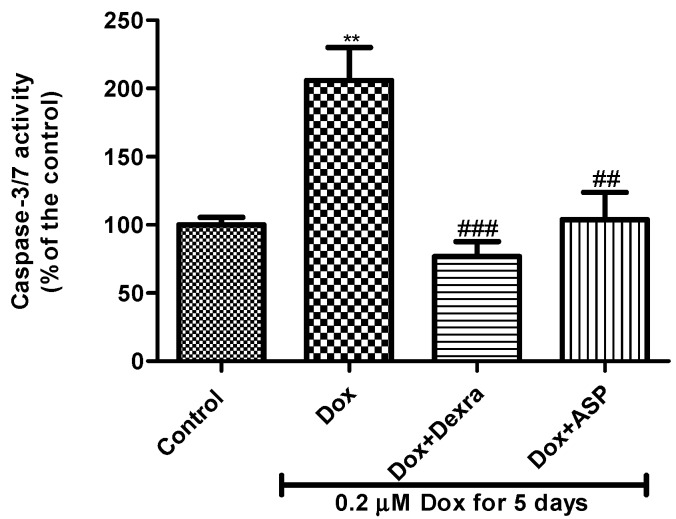
The effect of aspalathin (ASP) on caspase 3/7 activity on H9c2 cells treated with 0.2 µM Dox for 5 days. H9c2 cells were treated with 0.2 µM Dox alone or in combination with either 20 µM dexrazoxane (Dexra) or 0.2 µM ASP daily for 5 days. Control samples were exposed to media only. Results are expressed as the mean ± SEM of three independent biological experiments, each with three technical replicates (*n* = 9). ** *p* < 0.01 versus control and ^##^ *p* < 0.05 and ^###^ *p* < 0.01 versus Dox.

The Caspase-Glo^®^ 3/7 activity assay was used as a measurement of Dox-induced apoptotic cell death of H9c2 cardiomyoblasts. H9c2 cardiomyoblasts exposed to Dox displayed increased caspase-3/7 activity (205.9 ± 24.16%, *p* ≤ 0.01) when compared to the normal control (100%) (Figure 4). Co-treatment with Dox + Dexra (76.85 ± 10.92%, *p* ≤ 0.001) as well as Dox + ASP (103.9 ± 19.87%, *p* ≤ 0.01) significantly decreased Dox-induced apoptosis when compared to the Dox control group.

### 2.3. Transcriptional Regulation of Autophagy Gene in H9c2 Cardiomyoblasts

#### The Effect of ASP on the Activation of Autophagy Genes

To further investigate the role autophagy plays in Dox-induced cardiotoxicity, mRNA expression of genes essential for the activation (*Atg5*, *Atg7*, *Becln1 (Atg6)*, *BNIP3*, *Fox01*, and *AMPK*) and suppression (*mTOR*) of autophagy were assessed. The mRNA expression of autophagy-related genes were significantly decreased upon Dox treatment when compared to the untreated control, while ASP co-treatment were able to reverse this effect (Table 1).

### 2.4. The Effect of ASP Co-Treatment on the Efficacy of Dox to Increase Apoptosis in Caov-3 Ovarian Cancer Cells

#### 2.4.1. The Effect of ASP Co-Treatment on Caov-3 Cell Viability

**Figure 5:**
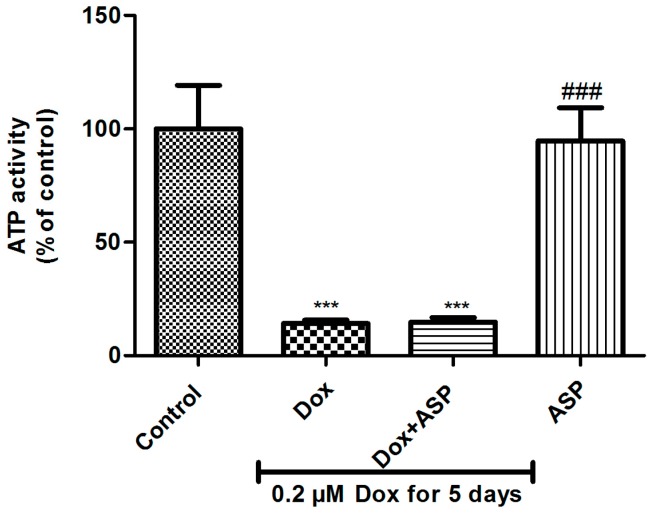
The effect of aspalathin (ASP) co-treatment on metabolic activity of Caov-3 cells exposed to Doxorubicin. Caov-3 cells were treated with either 0.2 µM Dox alone or co-treated in combination with 0.2 µM ASP, daily for 5 days. Control samples were exposed to either media only or 0.2 µM ASP for 5 days. Results are expressed as the mean ± SEM of three independent biological experiments, each with three technical replicates (*n* = 9). *** *p* ≤ 0.0001 versus control and ^###^ *p* ≤ 0.001 versus Dox.

ATP activity assay was used to assess cell viability and metabolic activity of Caov-3 cells co-treated with ASP (Figure 5). Interestingly, co-treatment with ASP was able to significantly reduced cell viability of Caov-3 cells when compared to the normal control (14.84 ± 1.9, compared to 100 ± 19.16, *p* < 0.01) (Figure 5). The observed effect was similar to treatment with Dox only (14.15 ± 1.5, compared to control 100.0, *p* < 0.01). Conversely, Caov-3 cells treated with ASP only had no effect on metabolic activity (94.75 ± 14.51) when compared to the normal control.

#### 2.4.2. The Effect of ASP Co-Treatment on the Expression of Pro-Apoptotic Proteins

**Figure 6:**
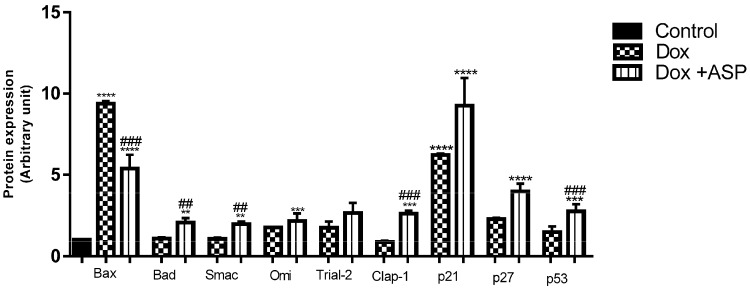
The effect of aspalathin (ASP) on pro-apoptotic protein expression in Caov-3 cells. Caov-3 ovarian cancer cells were treated with 0.2 µM Dox alone or in combination with 0.2 µM ASP daily for 5 days. Control samples were exposed to media only or ASP. Results are expressed as the mean ± SEM of three independent biological experiments, each with three technical replicates (*n* = 9). ** *p* ≤ 0.01, *** *p* ≤ 0.001 and **** *p* ≤ 0.0001 versus control and ^##^ *p* ≤ 0.01, and ^###^ *p* ≤ 0.001 versus Dox.

To determine if ASP co-treatment decreases the anti-cancer efficacy of Dox, various pro-apoptotic and tumour suppressor proteins were evaluated (Figure 6). Results obtained showed that Dox treatment was unable to increase the expression of pro-apoptotic proteins; Bad, Clap-1, Trial-2, Omi, and Smac when compared to the untreated control group. Co-treatment of Dox with ASP was able to induce apoptosis and significantly increase the expression of the latter genes. Furthermore, the expression of p27, p21, and p53 tumour suppressors that are known to inhibit the proliferation of cancer cells was significantly upregulated after Dox treatment and combination of Dox and ASP was able to enhance this effect.

## 3. Discussion

Autophagy can be defined as a process cells use to clear damaged proteins and organelles [19]. Although active in all cells, this process is only triggered in response to various types of cellular stresses and are thus selectively switched on [20]. Dox therapy induces such stress, resulting in cardiotoxicity through the induction of apoptosis, while decreasing autophagy. During increased stress, augmented autophagy is important to maintain cellular homeostasis and in the heart, this process has been associated with an improved heart pathology [21,22]. Furthermore, it has been suggested that pursuing genes that are activated upon autophagy could possibly be an effective therapeutic strategy to modulate Dox-induced cardiotoxicity. However, the precise molecular role autophagy plays in protecting the cells from Dox-induced cardiotoxicity remains poorly defined. The paradoxes stemming from identifying autophagy as a potential therapeutic target to impede Dox-induced cardiotoxicity remains the subject of extensive controversy, with autophagy having a dual function, pro-death or survival.

This adaptive response depends on the type of stimuli, duration of damage, experimental model used as well as experimental conditions [20,23]. In a study done by Sishi and colleagues (2013), the authors showed how manipulation of autophagy, through rapamycin-induced inhibition of mTOR, diminished the cardiotoxic effects of Dox on H9c2 myoblasts, as well as a tumour-bearing mouse model of acute Dox-induced cardiotoxicity [24]. Similarly, studies found that activation of Atg genes through increased AMPK medicated autophagy flux could ablate caspase-3 activity preventing Dox-induced cardiotoxicity [21,22]. Conversely, various investigators have also reported on the pro-death response of Dox, in which they showed that enhanced Dox-induced autophagy could lead to increased cardiotoxicity and subsequent apoptosis [25,26]. Thus, needless to say a complex interaction exists between apoptosis and autophagy, where autophagy and apoptosis may be triggered by a common upstream signal resulting in either a synergistic or antagonistic effect [27].

In recent years, it has been proposed that autophagy prevents Dox-induced apoptosis of cardiomyocytes by regulating the AMPK/mTOR/ATG signaling pathway [21,22]. In our study, we showed that a 0.2 µM of Dox daily for five days was able to enhance the expression of apoptotic proteins, but inhibited the expression of the autophagy-related genes. However, ASP was able to abrogate this effect without decreasing the efficacy of Dox. This result infers that an antagonistic effect exists between apoptosis and autophagy representing a biological mechanism for the pro-survival effect of ASP on Dox-treated H9c2 cardiomyoblasts.

**Figure 7:**
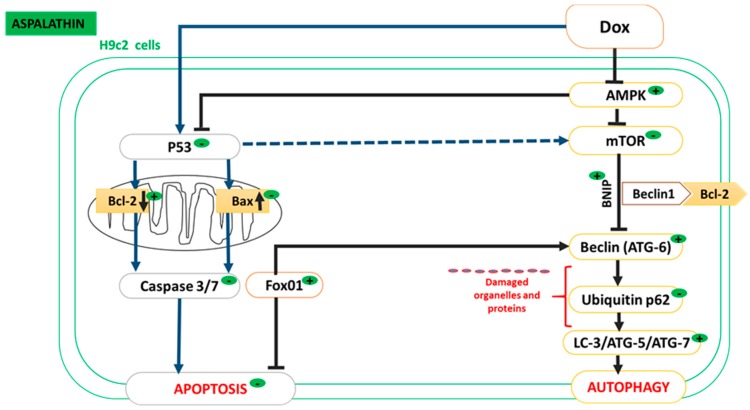
Aspalathin inhibits apoptosis and increase autophagy in H9c2 cells exposed to Doxorubicin (Dox). AMPK is a master regulator known to play an important role in energy homeostasis. Activation of AMPK attenuates Dox-induced activation of mTOR, restoring autophagy flux in H9c2 cells. Chronic exposure to Dox induces apoptosis through increased p53 and caspase-3/7 activity, a process known to be associated with cardiomyocyte loss. Activation of AMPK was able to inhibit p53 expression and enhances cardiomyocyte survival. AMPK, adenosine monophosphate-activated protein kinase ; Bax, Bcl-2-like protein 4; Bcl-2, B-cell lymphoma-2; ATG, autophagy-related genes; Cyt c, cytochrome C; mTOR, Mammalian target of rapamycin; LC3, Microtubule-associated protein 1A/1B-light chain 3 (LC3); P62, Nucleoporin p62.

To unravel the mechanisms whereby ASP increases autophagy and decreases apoptosis in H9c2 cardiomyoblasts, various genes within the autophagy process were investigated and the following mechanism are proposed (Figure 7). Autophagy entails five important steps, initiation, nucleation, elongation, maturation, and degradation (Kang et al 2011). We investigated the expression of two kinases, mTOR and its upstream regulator, AMPK, important in the suppression and activation of this process. Whereas, mTOR is a known inhibitor of autophagy, AMPK can initiate autophagy in an mTOR dependent or independent manner [28,29]. This study showed that Dox treatment inhibits autophagy through increased mTOR and decreased AMPK expression. However, ASP treatment increased the expression of AMPK without modulating mTOR, suggesting that AMPK might initiate autophagy in an mTOR independent manner through Atg1 (ULK1). However, Atg1 was not investigated in this study, and will form part of a follow up investigation [30].

Once autophagy is activated, Beclin1 (Atg6), a major regulator of autophagy, is recruited. Activation of Beclin1 requires the localization of autophagic proteins to form a pre-autophagosomal structure that will coordinate and regulate the nucleation process, a process central to the formation of the autophagosome. This process can be inhibited through the co-localization of Bcl-2 with Beclin1 in the endoplasmic reticulum [31]. Conversely, in the mitochondria, the nucleation process is activated when Bnip3 displaces Bcl-2 from Beclin1. This was confirmed by various studies which showed that increased expression of Bnip3 mediates upregulation of autophagy preventing the formation of the Beclin/Bcl-2 complex [32]. In this study, a decrease in mRNA expression of Bnip3 and Beclin1 were observed after Dox treatment. However, ASP was able to increase the expression of the latter autophagy initiation genes. This result confirms previous findings from our group showing that ASP is able to activate BNIP3 and Beclin1 (unpublished data).

Once recruited the nucleation process is initiated where the ubiquitin-binding transcription factor p62 binds to Atg genes, including Beclin1, to selectively recognize and engulf damaged and misfolded proteins. Once engulfed, p62 is selectively degraded with increased autophagy flux. In this study, a decrease in p62 has been observed with increased Atg gene expression. In addition, it has been demonstrated that the final execution of autophagy depends on the association between Atg5/Atg7 with LC3-II [33]. Once activated, Atg5/Atg7 and Beclin1/p53 associate with LC3-II to promote the maturation of the autolysosome and subsequently increase autophagy.

Interestingly, Atg5/Atg7 can also be activated by the transcriptional factor Fox01, a known inducer of autophagy. It has been reported that Fox01 requires phosphorylation by Akt to inhibit autophagy [34]. However, Fox01 can activate autophagy in an Akt independent manner, by directly activating Atg7. Although Akt was not investigated, we speculate that ASP might potentiate Fox01-induced autophagy by increasing the association of Atg5/Atg7 with LC3-II, a process indicative of decreased apoptosis.

In summary, this study demonstrates that while Dox induces cardiotoxicity and subsequent apoptosis, ASP co-treatment inhibits p53 in H9c2 cardiomyoblasts, leading to a decrease in apoptosis and an increase in autophagy. The increase in autophagy was associated with the activation of AMPK, an ideal strategy to prevent Dox-induced cardiotoxicity.

Lastly, in the present study we explored the effect of ASP + Dox co-treatment on the protein expression of pro-apoptotic proteins using a Caov-3 ovarian cancer cell line and observed that Dox + ASP co-treatment was more effective in increasing the expression of the pro-apoptotic proteins compared to Dox treatment alone. Thus, providing plausible evidence that ASP is able to attenuate Dox-induced cardiotoxicity through increased apoptosis and decreased autophagy without decreasing the apoptotic efficacy of Dox in ovarian cancer cells.

## 4. Materials and Methods

### 4.1. Aspalathin Preparation for Cell Culture

Synthetic aspalathin was obtained from High Force Research (ca. 98%, batch SZI-356-54) (Durham, UK) and stock solution was prepared as described previously [35]. The stock solution was then diluted in Dulbecco’s Modified Eagle’s Medium to a final working solution of 1 µM.

### 4.2. Doxorubicin (Dox) and Dexrazoxane (Dexra) Dose

In a study performed by Chacko and colleagues (2015), the authors showed that exposing H9c2 cells to varying doses of Dox (including 0.1–1.5 µM) for 3 days resulted in a decrease in metabolic activity from 80 to 43% [36]. Whereas unpublished data from our group showed that exposing H9c2 cells to 0.2 µM for 5 days showed significant cytotoxicity with a cell viability of 39%, thus Doxorubicin was used at 0.2 μM daily for a total of 5 days, as previously described by Goldswain [37]. The aforementioned study investigated the effect of an acute and chronic dose and found that treating cells with 1 µM of Dox for 24 h did not induced apoptosis, nor was caspase 3 activated. Interestingly, they found that treating cells with a concentration of 1 µM for a period of 120 h reduced cell viability and increased caspase 3 activity. Based on these findings, a dose of 0.2 µM Dox daily for 5 days was used in all subsequent experiments.

Dexrazoxane is the only FDA approved cardio-protective agent and was used at a dose of 100 μM (20 µM/day) for 5 days as per previously described method of Deng [38].

### 4.3. Cell Culture and Treatment of H9c2 Cardiomyoblasts and Caov3 Ovarian Carcinoma Cells with Aspalathin

Embryonic ventricular rat heart derived H9c2 cardiomyoblasts (ATCC, CRL-1446) and ovarian carcinoma Caov3 cells (kind gift from Dr. Mervin Meyer, University of Western Cape) were used for the study. Cells were cultured in Dulbecco’s Modified Eagle's medium, supplemented with 10% fetal bovine serum, 100 U/mL penicillin, and 100 mg/mL streptomycin under standard tissue culture conditions (37 °C in a water-saturated atmosphere of 5% CO_2_). The media was refreshed after 48 h and upon 80% confluency, cells were split and plated in either 6- or 96-well plates in DMEM (containing 10% fetal bovine serum) for 48 h. To induce cardiotoxicity, cells were exposed to 0.2 μM of Dox and treated either with 0.2 μM ASP or 20 µM Dexrazoxane (Dexra) daily for 5 days. Cells exposed to media only were used as a normal control.

### 4.4. ATP Assay 

Adenosine triphosphate (ATP) activity as a measurement of H9c2 and Caov3 cell cytotoxicity was done using the ViaLight™ plus kit according to manufacturer’s instructions (Lonza, Basel, Switzerland). Luminescence was quantified using a BioTek^®^ FLx800 plate reader and Gen 5^®^ software (BioTek Instruments Inc., Winooski, VT, USA).

### 4.5. Western Blot Analysis

After the predetermined experimental conditions, H9c2 cells were lysed in ice-cold lysis buffer (Pierce Biotechnologies, Rockford, CA, USA). The lysates were centrifuged and the supernatant collected. Thirty micrograms of protein lysates were denatured and loaded onto a 10% SDS-polyacrylamide gels (Bio-Rad, Hercules, CA, USA) and transferred to a polyvinylidene fluoride membrane (PVDF) (Bio-Rad, Hercules, CA, USA). The PVDF membrane containing the proteins of interest were blocked in 5% (*w*/*v*) non-fat milk in 1x Tris-buffered saline with Tween-20 at room temperature for 2 h. The membrane was then incubated overnight at 4 °C with the following primary antibodies from Cell Signaling (Cell Signaling, Danvers, MA, USA), anti- LC3-II (1:1000), SQSTM1/p62 (1:1000), anti-p53 (1:1000), anti-Bcl-2 (1:1000), anti-Bax (1:250), p-AMPK (Thr^172^) (1:800), mTOR (1:1000), and phospho mTOR (1:1000). Subsequently, the membranes were washed and incubated with the relevant horseradish peroxidase conjugated secondary antibodies for 90 min at room temperature. All proteins were normalized to β-actin (1:500) that was used as a loading control. Proteins were detected and quantified using a Chemidoc-XRS imager and Quantity One software (Bio-Rad Laboratories, Hercules, CA, USA).

### 4.6. Caspase 3/7 Activity

Caspase 3/7 activity was assessed using a Caspase-Glo^®^ 3/7 activity kit (Promega, Madison, WI, USA) according to the manufacturer’s instructions. Briefly, following the pre-determined experimental conditions, H9c2 cells were lysed in 350 µL ice cold lysis buffer. Twenty microliters of the cell lysates was then transferred to a 96-well plate. Thereafter, an equal amount of Caspase-Glo^®^ reagent (~20 µL) (Promega, Madison, WI, USA) was added to each well and the plate was incubated in the dark. After 30 min, luminescence was measured in a BioTek^®^ FLX 800 plate reader using Gen 5^®^ software. Results were normalized to pre-determined protein content using RC DC™ (Promega, Madison, WI, USA) protein assay.

### 4.7. mRNA Expression Analysis

Total RNA was extracted from H9c2 cells using TRIzol reagent. Briefly, H9c2 cells were homogenized using a TissueLyser (Qiagen, Hilden, Germany) at 25 Hz for 2 min according to the manufacturer’s instructions. RNA was purified using an RNeasy Mini kit (Qiagen, Germantown, MD, USA) and genomic DNA was removed using the Ambion Turbo DNase kit (Applied Biosystems, Austin, TX, USA) according to the manufacturer’s instructions. RNA samples were converted to cDNA using the High Capacity Reverse Transcription kit (Applied Biosystems, Austin, TX, USA) as recommended by the manufacturers. Quantitative RT-PCR was performed on an ABI 7500 Instrument (Applied Biosystems, Austin, TX, USA). The reaction mix was prepared by adding 5 µL Taqman universal PCR master mix 0.5 µL Taqman Gene Expression Assay (for, *Atg 5*, *Atg6*, *Atg7*, *mTOR*, *Foxo1*, *AMPK* and *Beclin1*), 1 ng of cDNA, and RNAse-free water to a final volume of 10 µL. Quantitative RT-PCR was conducted as follows: 50 °C for 2 min and 9 °C for 10 min, followed by 40 cycles of 95 °C for 15 s and 60 °C for 1 min. Gene expression data were normalized to hypoxanthine-guanine phosphoribosyltransferase (HPRT) and beta-actin (ActB).

### 4.8. Proteome Profiler Human Apoptosis Array

After the predetermined experimental conditions, Caov3 cells were lysed in ice-cold lysis buffer (Pierce Biotechnologies, Rockford, CA, USA). Protein content was determined using RC-DC assay according to manufacturer’s instructions (Bio-Rad, Hercules, CA, USA). Samples were diluted with phosphate buffered to obtain a final concentration of 250 μg protein in 125 μL. Thereafter, samples were loaded and hybridized to the Proteome Profiler™ Array Human Apoptosis Array (R&D Systems Inc., Minneapolis, MN, USA) according to manufacturer’s instructions. (R&D Systems Inc., Minneapolis, MN, USA). Proteins spots were detected and quantified using a Chemidoc-XRS imager and Quantity One software (Bio-Rad Laboratories, Hercules, CA, USA).

### 4.9. Statistical Analysis

Data were expressed as the mean ± standard error of mean (SEM) of three independent biological experiments with each experiment containing at least three technical replicates. GraphPad Prism software (GraphPad Software Inc., La Jolla, CA, USA) was used for calculation of one-way multivariate ANOVA, followed by a Tukey post hoc test or Student t-test where appropriate. A *p*-value of ≤0.05 was deemed as statistically significant.

## Figures and Tables

**Table 1:** mRNA expression analysis of autophagy genes in H9c2 cells.

*Genes*	*Dox*	*Dox + Dexra*	*Dox + ASP*
*Atg5*	↓1.81 ± 0.19 *	↑1.24 ± 0.24	↑1.18 ± 0.25 ^#^
*Atg7*	↓1.56 ± 0.2 *	↑1.02 ± 0.28 ^#^	↑1.06± 0.30 ^##^
*Beclin1*	↓1.61 ± 0.21 *	↑1.13 ± 0.25 ^#^	↑1.03± 0.27 ^#^
*BNIP3*	↓1.38 ± 0.23	↑1.04 ± 0.33	↑1.17± 0.4
*Fox01*	↓1.40 ± 0.24	↑1.14 ± 0.31	↑1.18± 0.32 ^#^
*AMPK*	↓1.72 ± 0.29	↑1.38 ± 0.41	↑1.2± 0.34
*mTOR*	↑1.30 ± 0.21	↓2.24 ± 0.16	↓1.39± 0.19

Legend to Figure: Arrows indicate fold difference relative to the control set as 1. ↓: Down regulated; ↑: Up regulated. Results are expressed as the mean ± SEM. * *p* < 0.05 versus control and ^#^ *p* < 0.05 and ^##^ *p* < 0.01 versus Dox.
